# A 50-year data-driven model of disability and lesion load trajectories in progressive multiple sclerosis

**DOI:** 10.1093/braincomms/fcae474

**Published:** 2024-12-27

**Authors:** Neil P Oxtoby, Frederik Barkhof

**Affiliations:** UCL Hawkes Institute and Department of Computer Science, University College London, London, UK; UCL Hawkes Institute and UCL Queen Square Institute of Neurology, University College London, London, UK; Department of Radiology and Nuclear Medicine, Amsterdam UMC, Vrije Universiteit, 1100 DD Amsterdam, The Netherlands

## Abstract

This scientific commentary refers to ‘A data-driven model of disability progression in progressive multiple sclerosis’, by Garbarino *et al*. (https://doi.org/10.1093/braincomms/fcae434).

**This scientific commentary refers to ‘A data-driven model of disability progression in progressive multiple sclerosis’, by Garbarino *et al*. (https://doi.org/10.1093/braincomms/fcae434).**

Multiple sclerosis is a disease with a protracted disease course where disease outcomes are difficult to predict. Most patients present with an initial relapsing–remitting disease course and may enter a secondary progressive stage of multiple sclerosis. A subset of patients with less inflammatory activity (and relapses) presents with a primary progressive multiple sclerosis (PPMS) onset, typically at a later age and more frequently with spinal cord symptoms. The fact that PPMS and secondary progressive stage of multiple sclerosis fare a similar course later, signifies that disease duration in PPMS is underestimated and is even more difficult to predict than in relapsing forms of multiple sclerosis, where early relapses mark disease onset. Once PPMS manifests clinically, there may be variable amounts of MRI abnormalities and other clinical findings such as cognitive impairment, signifying that the disease onset must have occurred some time ago. There now emerge opportunities to estimate the true disease onset better using machine learning/artificial intelligence.

Data-driven disease progression models^1^ have been developed and applied to reveal insights into neurodegenerative disease progression for over a decade. In multiple sclerosis, event-based models^2^ have leveraged cross-sectional data to estimate the sequence of neurodegeneration^3^ and clinical events.^4^ The clustering counterpart of event-based modelling has uncovered image-based subtypes of progressive multiple sclerosis that were predictive (*post hoc*) of treatment response in clinical trials.^5^ These approaches were designed for characterizing disease progression using cross-sectional data, but they are not well-suited for longitudinal data (without careful consideration).

Garbarino *et al.*^6^ applied the Gaussian Process Progression Model^7^ (GPPM) to a large international dataset to map out longitudinal trajectories of disability and brain pathology in PPMS. The GPPM is a multivariate Bayesian nonparametric longitudinal model that assumes a largely monotonic disease trajectory and uses a series of disease markers to move patients around a disease axis by simultaneously estimating mixed effects and a latent disease *time-shift*—thereby characterizing disease progression at both group and individual levels without being restricted by a predetermined time axis such as calendar age. Previously the GPPM has been applied in Alzheimer’s disease,^7^ exploring multimodal changes across scales in the brain. Here, Garbarino *et al.*^6^ trained a GPPM of PPMS using longitudinal data from three clinical trials, with external validation on data from a fourth clinical trial. Input features included multiple sclerosis disability scores and features from brain MRI.

From an average of 2 years of data from each of 1581 patients, the GPPM mapped out a 50-year disease course, giving a picture of *average* change across patient outcomes on a common disease time axis. Broadly speaking, early clinical disability on the expanded disability status scale (EDSS) preceded relatively slowly accumulating brain MRI abnormality and other clinical measures. The average EDSS worsening plateaued after 20–30 years, whereas the average 9-hole peg test (9HPT) and symbol digit modalities test (SDMT) each worsened more gradually, but all with high individual-level variability in patient trajectories (see Fig. 1 in the paper^6^).

Arguably one of the most interesting outputs of the model is the time-reparameterization (or time-*shift*) parameter, suggesting a difference between observed and predicted disease duration. Disease duration is notoriously difficult to determine in multiple sclerosis, particularly in PPMS because it lacks the early signs of inflammatory activity that signify disease onset in relapsing forms of multiple sclerosis. This materializes in Table 1 of the paper, where the model-designated (see below) ‘Slow Progressors’ have double the DD of the ‘Fast Progressors’ despite being a few years younger. It might be interesting to compare this model-estimated disease-duration gap with other work, e.g. using a brain-age paradigm on only brain MRI scans.^8^ We expect differences since Garbarino *et al.* additionally included clinical measures as model inputs, which adds an element of circularity when interpreting a model.

When we set our current definition of DD aside and instead look at how Garbarino *et al.* used their model’s time-shift parameter clinically—by designating Slow- and Fast-Progressor outlier subgroups in an external dataset—the results suggest that the so-called Fast Progressors tended to be older at symptom onset (38.5 versus 35 years), male (61% versus 49%), carrying considerably higher brain MRI lesion load, and have higher baseline EDSS (6.0 versus 3.5). Subsequent survival analyses confirmed that Fast Progressors tended to have a poorer prognosis (as might be, expected from with the baseline characteristics). Importantly, the authors used sensible benchmarking^9^ of their model-based DD predictions against clinical DD (which was shown not be a relevant predictor).

Limitations included identifiability of the model, which relies upon (assumes) monotonicity of the population level trajectories. In principle, this limits the applicability of a GPPM to only the progressive forms of multiple sclerosis. It is also interesting to compare with an approach that models variability in an orthogonal manner: Subtype and Stage Inference^10^ (SuStaIn). The GPPM uses mixed-modelling to infer continuous temporal trajectories, whereas SuStaIn finds disease progression sequence clusters (subtypes). The authors switched off SuStaIn’s clustering feature to explore only a single sequence, which showed (in Supplementary Fig. 1) a consistent pattern of abnormality to the GPPM. While reassuring, this feels like a missed opportunity to explore whether the individual-level variability might be modelled well by disease progression subtypes, which have been shown to predict treatment response.^5^

In summary, this is the first application of a longitudinal data-driven disease progression model in multiple sclerosis that mapped out 50 years of clinical disability and brain pathology. While this study helps us to better understand what marks the (onset of) progression of PPMS, much remains unexplained due to unavailability of key biological features. This might include markers of chronic active inflammation, as the authors suggest, but also markers of spinal cord damage which is the most frequent site of pathology in PPMS. For example, cord lesion number and cord atrophy have been shown to be important predictors in PPMS and should be considered in future modelling work. If available, more sensitive measures of bowel/bladder function might also be considered. The most important lesson from this work is that disease duration in multiple sclerosis is grossly underestimated and that novel approaches (here the GPPM) can determine a DD-gap that is relevant for prognosis and yields insight into the ‘preclinical’ stages of PPMS.

## Data Availability

No new data were generated or analysed in support of this commentary.

## References

[1] Young AL , Oxtoby NP, Garbarino S, et al Data-driven modelling of neurodegenerative disease progression: Thinking outside the black box. Nat Rev Neurosci. 2024;25:111–130. 38191721 10.1038/s41583-023-00779-6

[2] Fonteijn HM , Modat M, Clarkson MJ, et al An event-based model for disease progression and its application in familial Alzheimer’s disease and Huntington’s disease. NeuroImage. 2012;60(3):1880–1889. 22281676 10.1016/j.neuroimage.2012.01.062

[3] Eshaghi A , Marinescu RV, Young AL, et al Progression of regional grey matter atrophy in multiple sclerosis. Brain. 2018;141(6):1665–1677. 29741648 10.1093/brain/awy088 PMC5995197

[4] Dekker I , Schoonheim MM, Venkatraghavan V, et al The sequence of structural, functional and cognitive changes in multiple sclerosis. NeuroImage Clin. 2021;29:102550. 33418173 10.1016/j.nicl.2020.102550 PMC7804841

[5] Eshaghi A , Young AL, Wijeratne PA, et al Identifying multiple sclerosis subtypes using unsupervised machine learning and MRI data. Nat Commun. 2021;12(1):2078. 33824310 10.1038/s41467-021-22265-2 PMC8024377

[6] Garbarino S , Tur C, Lorenzi M, et al A data-driven model of disability progression in progressive multiple sclerosis. Brain Commun. 2024:10.1093/braincomms/fcae434. PMC11704797 39777254

[7] Lorenzi M , Filippone M, Frisoni GB, Alexander DC, Ourselin S. Probabilistic disease progression modeling to characterize diagnostic uncertainty: Application to staging and prediction in Alzheimer’s disease. NeuroImage. 2019;190:56–68. 29079521 10.1016/j.neuroimage.2017.08.059

[8] Pontillo G , Prados F, Colman J, et al Disentangling neurodegeneration from aging in multiple sclerosis using deep learning. Neurology. 2024;103(10):e209976. 39496109 10.1212/WNL.0000000000209976 PMC11540460

[9] Marinescu RV , Oxtoby NP, Young AL, et al The Alzheimer’s Disease Prediction Of Longitudinal Evolution (TADPOLE) challenge: Results after 1 year follow-up. Mach Learn Biomed Imaging. 2021;1(December 2021):1–10.

[10] Young AL , Marinescu RV, Oxtoby NP, et al Uncovering the heterogeneity and temporal complexity of neurodegenerative diseases with Subtype and Stage Inference. Nat Commun. 2018;9(1):4273. 30323170 10.1038/s41467-018-05892-0 PMC6189176

